# CTRP1 Aggravates Cardiac Dysfunction Post Myocardial Infarction by Modulating TLR4 in Macrophages

**DOI:** 10.3389/fimmu.2021.635267

**Published:** 2021-05-07

**Authors:** Yang Gu, Xiao Hu, Pei-Bing Ge, Yu Chen, Shen Wu, Xi-Wen Zhang

**Affiliations:** Department of Cardiology, The Affiliated Huaian No.1 People’s Hospital of Nanjing Medical University, Huai’an, China

**Keywords:** myocardial infarction, cardiac dysfunction, CTRP1, TLR4, macrophages

## Abstract

CTRP1 (C1q/TNF-α [tumour necrosis factor-α]-related protein 1), an adiponectin paralog, is associated with diabetes and adverse events in cardiovascular disease. However, its effect on cardiac function post myocardial infarction (MI) is unclear. Our study aimed to explore the role of CTRP1 in cardiac function post MI. CTRP1 global knockout mice were subjected to left anterior descending ligation to establish the MI model. C57BL6J mice were also administered recombinant CTRP1 protein (200 μg/kg) 7 days post MI. As a result, mice with CTRP1 deficiency exhibited an increased survival rate, a reduced infarct area, improved cardiac function and decreased inflammation and oxidative stress levels at 4 weeks post MI compared with those of mice receiving the CRTP1 injection, whose conditions deteriorated. However, cardiomyocytes with either CTRP1 silencing or CTRP1 treatment showed few differences in inflammation and oxidative stress levels compared with those of the control under hypoxic conditions. The activation of macrophages isolated from CTRP1-deficient mice was decreased in response to interferon-γ, while CTRP1 enhanced the activation of macrophages in response to interferon-γ. Macrophage scavengers and clodronate liposomes antagonized the effects of CTRP1 injection in mice. We also found that CTRP1 regulated macrophage activation *via* adiponectin receptor 1, which binds to TLR4 on the macrophage membrane. TLR4 knockout also antagonized the effects of the CTRP1 protein on mice with MI. Taken together, these data indicate that CTRP1 supresses cardiac function post MI *via* TLR4 on macrophages. Targeting CTRP1 may become a promising therapeutic approach to cardiac dysfunction post MI.

## Introduction

During the acute phase of myocardial infarction (MI), cardiomyocyte necrosis leads to the initiation of the inflammatory response (1). “In this stage, monocytes from peripheral blood and heart tissue are gathered (2). Damage-associated molecular patterns (DAMPs) from necrotic cardiomyocytes bonded to specific pattern recognition receptors (PRRs) on the surface of inflammatory cells and other surviving cardiomyocytes cause an inflammatory cascade (3). This inflammation is beneficial in the early stage of MI, as it accelerates the clearance of dead cardiomyocytes and promotes cardiac fibrosis to maintain cardiac structure and function (4). During the late stage (more than 14 days post MI), sustained inflammation and fibrosis cause cardiac remodelling and heart failure (5). Many PRRs, such as Toll-like receptor (TRL) and NOD-like receptor (NLR) family members, account for the persistence of inflammation (6, 7), and targeting these receptors may be a promising therapeutic strategy for cardiac remodelling post MI.

CTRP1 derives from a family of adiponectin paralogues, and many CTRP family members are involved in cardiovascular diseases in recent years. Circulating CTRP1, CTRP9, and CTRP12 levels were found to be elevated in type 2 diabetes mellitus cases and to regulate glucose lipid metabolism and insulin resistance (8, 9). Serum CTRP1 and CTRP3 levels were found to be closely associated with adverse events in coronary artery disease (10–12). In an animal study, CTRP1 was reported to stimulate aldosterone production and was adversely associated with hypertension (13, 14). However, Jiang W et al. recently reported that CTRP1 could prevent sepsis-induced cardiomyopathy (15), which contradicts previous studies. Based on the studies above, we hypothesize that CTRP1 may play a role in modulating cardiac remodelling post MI, and we herein used CTRP1 global knockout (KO) mice and recombinant CTRP1 protein to elucidate the functional role of CTRP1 in cardiac remodelling post MI.

## Methods

### Animals

CTRP1-KO mice were purchased from Cyagen Biosciences (Guangzhou, China, KOCMP-07979). C57BL6J mice (male) were purchased from Beijing Huafukang Biology Co., Ltd (Beijing, China). TLR4-KO mice were purchased from Jackson Laboratory (stock no: 029015). C57BL6J mice purchased from the Chinese Academy of Medical Sciences (Beijing, China) were subjected to intraperitoneal injection of recombinant human CTRP1 full-length protein (Abcam, ab151376; 200 μg/kg) every other day from days 7 to 28 post MI). For the macrophage clearance experiment, mice were subjected to intravenous injection of clodronate liposomes (CLs; 150 mL; 5 mg/mL; Liposoma, The Netherlands) at 10- and 14-days post MI. For the TLR4 deficiency experiment, TLR4-KO mice were subjected to intraperitoneal injection of recombinant human CTRP1 (200 μg/kg, from 7 days to 28 days post MI). All animal experiments were approved by the Institutional Animal Care and Use Committee of Huai’an First People’s Hospital, Nanjing Medical University (Huai’an, China).

### Animal Model

#### Left Coronary Artery Ligation Surgery

The left coronary artery ligation surgery (LAD) was conducted according to previously published guidance. In short, after anaesthetization, the mouse chest was opened on the left side between the third and fourth intercostal spaces. After opening the pericardium, the proximal descending branch of the LAD was ligated with 7~0 silk thread; the LAD was not ligated in the sham group. The survival rate of the mice post MI was evaluated.

### Echocardiographic Evaluation

Transthoracic echocardiography was performed as previously described (16). Isoflurane (1.5%) was used to anaesthetize the mice, and echocardiography was performed with a 10-MHz linear-array ultrasound transducer to obtain M-mode echocardiography data. The left ventricle (LV) end-diastolic dimension (LVEDd) and LV end-systolic dimension (LVESd) were obtained, and the LV ejection fraction (LVEF) and LV fractional shortening (LVFS) values were calculated. A total of 10 mice from each group were subjected to transthoracic echocardiography.

### Haematoxylin and Eosin (H&E), Immunohistochemistry Staining, Immunofluorescence Staining

Triphenyltetrazolium chloride (TTC, 1%, Sigma, USA) staining was used to evaluate the MI area and morphological changes in the heart. For the infarct area calculation, Image-Pro Plus 6.0 was used to analyse 6 sections from each heart and 6 hearts from each group. Macrophages were subjected to immunohistochemistry staining for CD68. After dehydration, antigen repair was conducted at a high temperature and pressure, and the sections were sealed with 8% goat serum. The heart sections were incubated with an anti-CD68 antibody (Abcam, 1:100 dilution) and then with an anti-rabbit horseradish peroxidase (HRP) reagent (Gene Tech, Shanghai, China). A peroxide-based substrate DAB kit (Gene Tech, Shanghai, China) was used for coloration. Macrophages were subjected to immunohistochemistry staining for F4/80, NOS2, and CD206 to detect M1 and M2 positive cells. The heart sections were incubated with an anti- F4/80, NOS2, and/or CD206 antibody (Abcam, 1:100 dilution). Secondary antibody goat anti-mouse/rabbit IRdye 800CW (LI-COR) were used. The nuclear was stained with DAPI. We counted the number of F4/80, NOS2, or F4/80, CD206 positive cells for each group (10 field for each heart).

### ELISA Detection of Inflammatory Cytokines

Tumour necrosis factor α, interleukin (IL)-1, and IL-6 from mouse hearts as well as cardiomyocytes and macrophages were detected with ELISAs purchased from BioLegend (430901, 432604, 431304). An ELISA instrument (Synergy HT, BioTek, United States) was used to measure the absorbance.

### Oxidative Stress Assessment

The activities of manganese superoxide dismutase, superoxide dismutase 2 (MnSOD), nicotinamide adenine dinucleotide phosphate (NADPH) oxidase, and glutathione peroxidase (Gpx) as well as the levels of malondialdehyde (MDA) in heart tissues and cardiomyocytes were detected by corresponding kits purchased from Beyotime (Shanghai, China) according to the manufacturers’ instructions.

### Cardiomyocyte and Macrophage Isolation and Culture

Neonatal rat cardiomyocyte (NRCM) culture was performed as previously described (16). Briefly, the hearts of Sprague-Dawley rats (1-3 days old) were quickly removed, and ventricles were preserved and digested with 0.125% trypsin-EDTA (Gibco) 4 times for 15 min each time. Digestion was halted with DMEM-F12 supplemented with 15% foetal bovine serum (FBS, Gibco, USA). After 5 digestion reactions, the cells were collected and incubated in a 100-mm dish with DMEM-F12 supplemented with 15% FBS. After 90 minutes, the cell culture medium was collected, and NRCMs in the upper layer of the cell medium were removed and seeded onto a 6-well plate to exclude the non-cardiac myocytes adhered to the bottom of the 100-mm dish. NRCMs were identified by α-actin staining. The NRCM hypoxia model was established in a Biospherix C-Chamber (model C-274, Biospherix, Redfield, NY, United States). NRCMs in the control group were cultured in 5% CO_2_ and 95% air. NRCMs were treated with 4 μg/mL human recombinant CTRP1 for 24 h during the hypoxia process to assess the effect of CTRP1 on cardiomyocytes. NRCMs were transfected with CTRP1 siRNA (RiboBio, China) to knockdown CTRP1.

Primary bone marrow-derived macrophages were isolated from the femurs and tibias of CTRP1-KO and CTRP1-WT male mice (6-8 weeks) and cultured in DMEM-F12 supplemented with 5% FBS, L-glutamine (5 mmol/litre, Sigma), and recombinant macrophage colony-stimulating factor (MCSF, 25 ng/ml, Peprotech) at 37 °C in 5% CO_2_. On day 6, the cells were stimulated with MCSF (25 ng/ml) and granulocyte-MCSF (GMCSF, 50 ng/ml, Peprotech) to induce differentiation. Then, macrophages were stimulated with interferon γ (IFN-γ) (10 ng/ml, Peprotech) and lipopolysaccharide (LPS) (100 ng/ml, Sigma) to induce pro-inflammatory activation (17) and treated with CTRP1 (4 μg/mL) for 24 h.

### Adult Mouse Cardiomyocyte/Cardiac Macrophage Isolation

Adult mouse cardiomyocytes were obtained from mice post MI *via* the Langendorff method according to a previous study (18). Heparin (100 U) was intraperitoneally injected into the mice, and their hearts were removed and hung on a modified Langendorff perfusion system *via* the aorta. A circulating enzyme digestion solution was flowed through the heart at a speed of 3 mL/min for 15–20 min at 37°C. When the heart became pale and flaccid, it was removed, and the ventricular tissue was cut with forceps and gentle pipetting. Finally, the isolated cells were filtered and resuspended in 12 mL of stopping buffer containing 1 mM CaCl_2_. Then, the cells were plated on laminin-coated 35-mm culture dishes in MEM containing 20 mM HEPES, 4 mM NaHCO_3_, 0.1 mg/mL bovine serum albumin, 2 mM L-glutamine, 1× insulin transferrin-selenium supplement (Sigma), and 10 mM 2,3-butanedione monoxime (BDM).

Adult mouse hearts were removed from mice post MI, diced into small pieces and digested with 1× PBS supplemented with 100 μg/mL collagenase IV (19). After five digestions, the samples were filtered through a 100 μm nylon cell strainer, centrifuged, and resuspended. Erythrocytes were removed with red blood cell lysis buffer (600 μL), after which the cells were washed with 10 mL of FACS buffer and blocked with an anti-CD16/CD32 Ab (1:100). Then, the cells were incubated with an antibody mixture [CD45-PerCPCy5.5 (2 μg/ml), CD11b-PECy7 (2 μg/ml), F4/80-PE (6 μg/ml), Ly6C-APC (4 μg/ml), MHCII-BV605 (0.67 μg/ml), CCR2-APC (5 μg/ml)] in FACS buffer (all obtained from BioLegend). Flow cytometry analysis was performed on BD FACSCanto II flow cytometer (BD Biosciences, Franklin Lakes, NJ, USA), and the cells were then lysed for western blotting.

### CTRP1 Concentration Detection

CTRP1 concentration in serum, adipose tissue and heart tissue was measured using a commercially available enzyme-linked immunosorbent assay (ELISA) kit (BioVendor, Inc., Czech Republic; Catalog number: RD191153100R) according to manufacturer’s protocol.

### Western Blot and Co-Immunoprecipitation

Total protein was isolated from heart tissues, NRCMs and macrophages and then subjected to SDS-PAGE (50 μg per sample). After transfer onto Immobilon membranes (Millipore, Billerica, MA, USA), proteins were incubated overnight at 4°C with primary antibodies against CTRP1, adiponectin R1 (Adipo R1), and Adipo R2 purchased from Abcam (1:1000 dilution) and TLR4 and GAPDH purchased from (Cell Signaling Technology (1:1000 dilution). Blots were developed with enhanced chemiluminescence (ECL) reagents (Bio-Rad, Hercules, CA, USA) and captured by a ChemiDoc MP Imaging System (Bio-Rad). GAPDH served as an internal reference protein.

Bone marrow-derived macrophages were cotransfected with psicoR-HA-TLR4 and psicoRFlag-CTRP1 or psicoRFlag-Adipo R1. The macrophage lysates were treated with a Protein G Plus/Protein A agarose suspension (Santa Cruz, CA, USA) and then incubated with antibodies against HA or Flag (Proteintech, USA). An agarose suspension of Protein G Plus/Protein A was added once again, and immunoprecipitants were subjected to SDS-PAGE electrophoresis.

### Statistical Analysis

All data are expressed as the mean ± SD. Differences among groups were analysed by two-way analysis of variance followed by Tukey’s *post hoc* test. Comparisons between two groups were analysed by an unpaired Student’s t-test. P values less than 0.05 indicated statistical significance.

## Results

### CTRP1 Expression Is Increased in Humans and Mice Post MI

**Figure 1:**
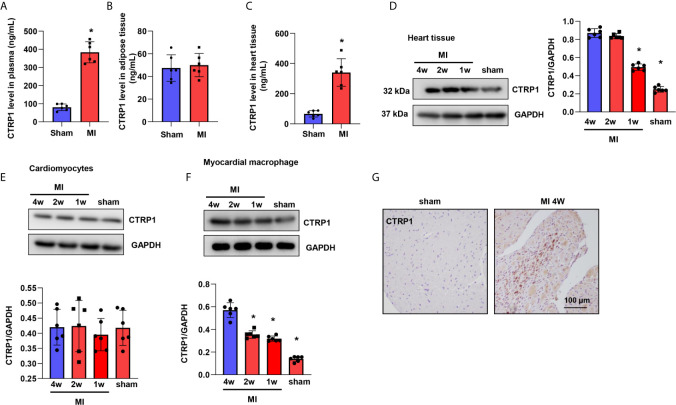
CTRP1 expression is increased in humans and mice post MI. **(A)** CTRP1 protein level in mouse plasma 4 weeks after MI detected by Elisa assay (n=6). **(B)** CTRP1 protein level in mouse peritoneal adipose tissue 4 weeks after MI detected by Elisa assay (n=6). **(C)** CTRP1 protein level in mouse heart tissue 4 weeks after MI detected by Elisa assay (n=6). **(D)** The CTRP1 protein expression in mouse hearts post MI (n=6). **(E)** The CTRP1 protein expression in cardiomyocytes isolated from mouse hearts post MI (n=6). **(F)** The CTRP1 protein expression in macrophages isolated from mouse hearts post MI (n=6). **(G)** CTRP1 staining in mouse hearts post MI (n=5). *P<0.05 *vs.* the sham group.

We first evaluated the protein of CTRP1 level in mice plasma, heart tissue as well as adipose tissue. As shown in **Figures 1A–C**, we found that the level of CTRP1 in mice plasma was elevated 4 weeks after MI (**Figure 1A**). No difference was observed for CTRP1 level in adipose tissue in mice at MI group and control group (**Figure 1B**). CTRP1 in heart tissue was sharply elevated in MI group (**Figure 1C**). We also detected CTRP1 protein level with western blot in heart tissue and different cell types. In mouse hearts post MI and found that the CTRP1 protein level was increased from days 7 to 28 post MI compared to that in sham mouse hearts (**Figure 1D**). We then isolated cardiomyocytes from mouse hearts post MI and examined CTRP1 protein expression, which did not change from that in the sham group (**Figure 1E**). We also isolated cardiac fibroblast, The CTRP1 protein level in cardiac fibroblast was undetectable. Since CTRP1 is also expressed in macrophages, we isolated cardiac macrophages from mice post MI; the CTRP1 protein expression trend was consistent with that in mouse hearts, increasing from day 7 to day 28 post MI (**Figure 1F**). We then stained for CTRP1 in mouse hearts at 28 days post MI, revealing that the protein was localized mainly in inflammatory cells in the peri-infarct mouse heart area (**Figure 1G**).

### CTRP1 Deficiency Reduces Cardiac Dysfunction Post MI

**Figure 2:**
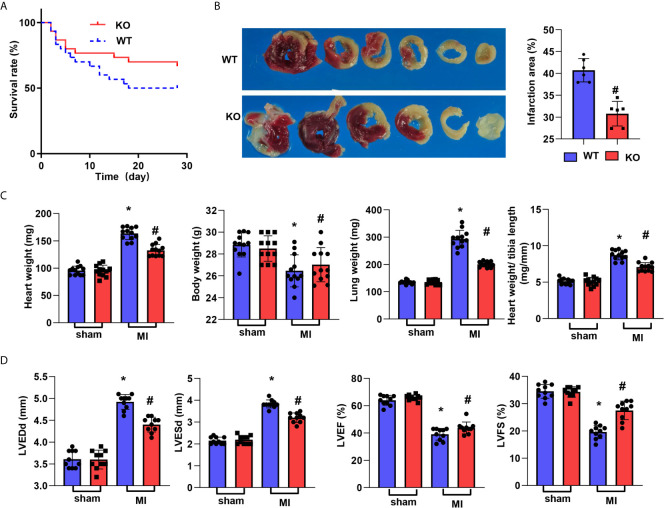
CTRP1 deficiency reduces cardiac dysfunction post MI. **(A)** The survival rate of CTRP1 knockout mice at 28 days post MI (n=30 per group). **(B)** TTC staining and quantification of the infarct area (n=6). **(C)** Heart, body, and lung weights in mice at 28 days post MI (n=12). **(D)** Echocardiography of mice at 28 days post MI (n=10). *P<0.05 *vs.* the WT-sham group. ^#^ *P*<0.05 *vs.* the WT-MI group.

CTRP1-KO mice were subjected to LAD, and their death rate was lower than that of mice in the WT group (67.6% *vs.* 50%, **Figure 2A**). The infarct size at 28 days post MI was smaller than that in the WT group (**Figure 2B**). The body weights of mice in the WT group post MI were lower than those of mice in the corresponding sham group, but there were no body weight differences between the WT and KO groups post MI. The wet heart weights were sharply increased in the MI group and decreased in the KO-MI group compared with the WT-KO group. The lung weights, representing lung pulmonary oedema, were lower in the KO-MI group than in the WT-KO group (**Figure 2C**). We then assessed cardiac function, and at 28 days post MI, both the LVEDd and LVESd were increased in MI mouse hearts compared to those of sham mice and were smaller in the KO-MI group than in the WT-KO group (**Figure 2D**). The LVEF and LVFS were also decreased in the MI group but were both higher in the KO-MI group than in the WT-KO group (**Figure 2D**).

### CTRP1 Deficiency Suppresses Cardiac Inflammation and OS Post MI

**Figure 3:**
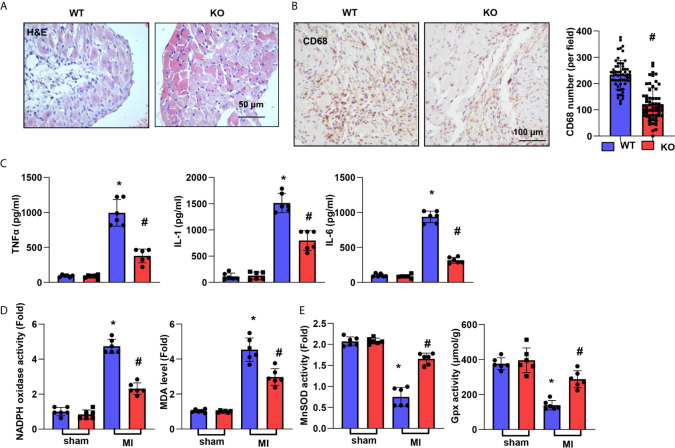
CTRP1 deficiency suppresses cardiac inflammation and OS post MI. **(A)** H&E staining. **(B)** CD68 staining and quantification of the results in CTRP1 knockout mice at 28 days post MI (n=6 per group). **(C)** Inflammatory cytokine levels in heart tissue post MI (n=6 per group). **(D**, **E)** Oxidase and anti-oxidase activities and MDA levels in heart tissue post MI (n=6 per group). *P<0.05 *vs.* the WT-sham group. ^#^ *P*<0.05 *vs.* the WT-MI group.

Inflammation was assessed by H&E and CD68 staining. As shown in **Figure 3A**, the inflammatory cell infiltration in the peri-infarct area at 28 days post MI was reduced in the CTRP1-KO group compared with the WT group. The number of CD68-labelled macrophages in heart tissue was remarkably reduced in the KO group compared with the WT group (**Figure 3B**). The cardiac levels of the inflammatory cytokines TNFα, IL-1, and IL-6 post MI were much higher than those in the sham hearts and lower in CTRP1-KO mouse hearts than in WT hearts (**Figure 3C**). OS in heart tissue was assessed by an oxidase/anti-oxidase system. The levels of NADPH oxidase and MDA were lower in KO mouse hearts than in WT mouse hearts post MI, while MnSOD and Gpx activity was higher in KO mouse hearts than in WT mouse hearts post MI (**Figure 3D**).

### Human Recombinant CTRP1 Amplifies Cardiac Dysfunction Post MI

**Figure 4:**
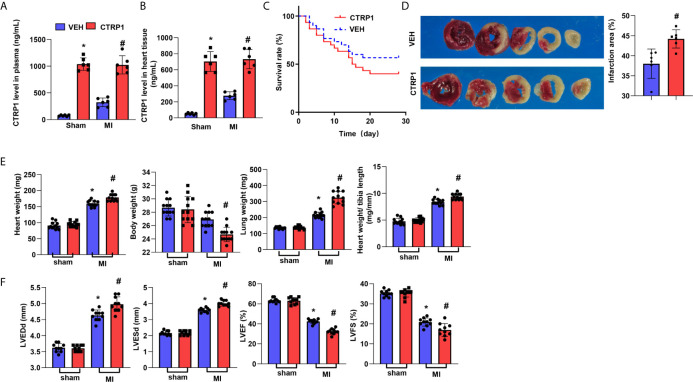
Human recombinant CTRP1 amplifies cardiac dysfunction post MI. **(A)** Human CTRP1 protein level in mouse plasma 3 weeks after injection detected by Elisa (n=6). **(B)** Human CTRP1 level in mouse heart tissue 3 weeks after injection detected by Elisa (n=6) **(C)** The survival rate of mice receiving the human recombinant CTRP1 protein at 28 days post MI (n=30 per group). **(D)** TTC staining and quantification of the infarct area (n=6). **(E)** Heart, body, and lung weights of mice at 28 days post MI (n=12). **(F)** Echocardiography of mice at 28 days post MI (n=10). *P<0.05 *vs.* the VEH-sham group. ^#^ *P*<0.05 *vs.* the VEH-MI group.

We hypothesized that the CTRP1 protein accelerates cardiac dysfunction post MI. Human CTRP1 was injected into C57BL6J mice at 7 days post MI. As shown in **Figure 4A**, human CTRP1 level in mice plasma was elevated in both CTRP-1-sham group and CTRP1-MI group after 3 weeks of CTRP1 injection. Human CTRP1 level in mice heart tissue was also elevated in both CTRP-1-sham group and CTRP1-MI group after 3 weeks of CTRP1 injection (**Figure 4B**). The death rate of mice receiving the CTRP1 injection was increased compared with that of the vehicle group (VEH, received an equal volume of normal saline) (**Figure 4C**). The infarct size was also increased in mice receiving the CTRP1 injection (**Figure 4D**). The mouse body weights were unchanged in the VEH-MI group compared with the VEH-sham group but sharply decreased in the CTRP1-MI group. The heart and lung weights were also sharply increased in the CTRP1-MI group compared with the VEH-MI group (**Figure 4E**). Cardiac dysfunction was obviously diminished in the CTRP1-MI group, with increased LVEDd and LVESd values and decreased LVEF and LVFS values compared with those in the VEH-MI group (**Figure 4F**). Thus, our extra injection of CTRP1 confirm the deteriorating effects of human CTRP1 on cardiac remodelling post MI.

### Human Recombinant CTRP1 Enhances Cardiac Inflammation and OS Post MI

**Figure 5:**
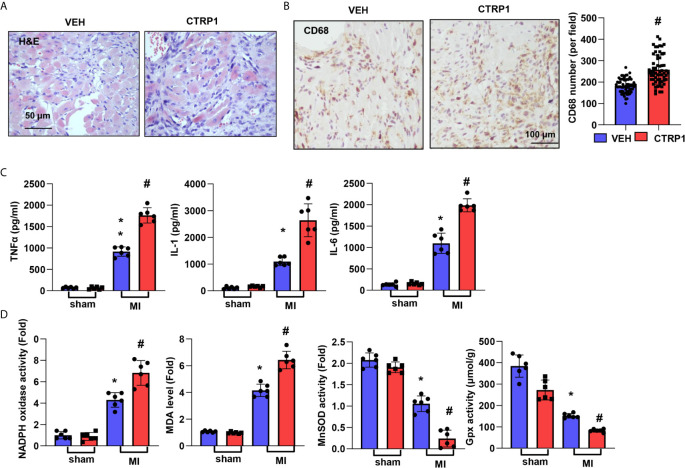
Human recombinant CTRP1 enhances cardiac inflammation and OS post MI. **(A)** H&E staining. **(B)** CD68 staining and quantification of the results in mice receiving the human recombinant CTRP1 protein at 28 days post MI (n=6 per group). **(C)** Inflammatory cytokine levels in heart tissue post MI (n=6 per group). **(D)** Oxidase and anti-oxidase activities and MDA levels in heart tissue post MI (n=6 per group). *P<0.05 *vs.* the VEH-sham group. ^#^ *P*<0.05 *vs.* the VEH-MI group.

Inflammatory cell infiltration in the peri-infarct area at 28 days post MI was increased to a greater extent in mice injected with the recombinant CTRP1 protein than in mice injected with the VEH (**Figure 5A**). The number of CD68-labelled macrophages in heart tissue was remarkably increased in the CTRP1-MI group compared with the VEH-MI group (**Figure 5B**). The levels of the inflammatory cytokines TNFα, IL-1, and IL-6 post MI were much higher in mouse hearts in the CTRP1-MI group than in those of the VEH-MI group (**Figure 5C**). The levels of NADPH oxidase and MDA were higher in the CTRP1-MI group than in the VEH-MI group, while the MnSOD and Gpx activities were lower in the CTRP1-MI group than in the VEH-MI group (**Figure 5D**).

### Macrophages Account for the Effect of CTRP1 on Cardiomyocytes

**Figure 6:**
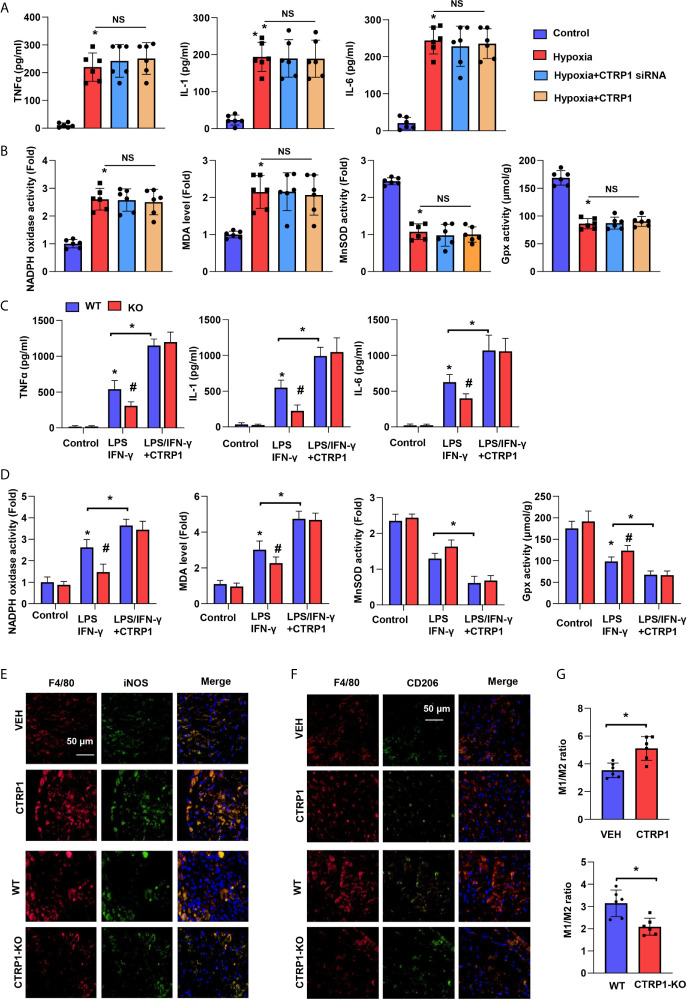
Macrophages account for the effect of CTRP1 on cardiomyocytes. **(A)** Inflammatory cytokine levels in NRCMs transfected with CTRP1 siRNA or the human recombinant CTRP1 protein under hypoxia (24 h). **(B)** Oxidase and anti-oxidase activities and MDA levels in NRCMs in the indicated groups. *P<0.05 *vs.* the control group. **(C)** Primary bone marrow-derived macrophages isolated from CTRP-/- mice treated with LPS/IFN-γ and the CTRP1 protein for 24 h. Inflammatory cytokine levels in the indicated groups. **(D)** NRCMs were cultured with the macrophage supernatants in the indicated groups. Oxidase and anti-oxidase activities and MDA levels in NRCMs. *P<0.05 *vs.* the WT control group. ^#^ *P*<0.05 *vs.* the WT-LPS+IFN-γ group. All the *in vitro* experiments were repeated 3 times. NS, not significant. **(E)** Representative image of immunofluorescence staining for F4/80 and NOS2, or F4/80 and CD206 in CTRP1-KO mice post MI. **(F)** Representative image of immunofluorescence staining for F4/80 and NOS2, or F4/80 and CD206 in CTRP1 injected mice post MI. **(G)** Quantification result of F/480^+^/NOS2^+^ to F/480^+^/CD206^+^macrophage ratio in the indicated group (n=6). *P<0.05 *vs.* the WT/VEH group.

We used an *in vitro* hypoxia model to investigate the role of CTRP1 in cardiomyocytes. NRCMs were exposed to hypoxia and transfected with CTRP siRNA or treated with CTRP1 protein for 24 h prior to assessing the levels of inflammation and OS. NRCMs in the hypoxia group exhibited increased levels of the inflammatory cytokines TNFα, IL-1, and IL-6 compared with those in control group cells. Nevertheless, the cytokine trends were affected by neither CTRP1 siRNA nor recombinant human CTRP1 protein treatment (**Figure 6A**). Similar to the OS level, NRCMs in the hypoxia group exhibited stronger NADPH oxidase activity, lower MDA levels and weaker MnSOD and Gpx activity than control group cells. Neither CTRP1 siRNA nor recombinant human CTRP1 protein treatment altered these changes under hypoxic conditions (**Figure 6B**).

Primary bone marrow-derived macrophages were isolated from CTRP1-KO mice and stimulated with LPS/IFN-γ and recombinant human CTRP1. The cytokine release and OS of macrophages in KO mice were impaired compared with those from WT mice upon stimulation with IFN-γ (**Figures 6C, D**). After recombinant human CTRP1 treatment, the cytokine release and OS of macrophages in KO mice did not differ from those in WT mice. We also detected the effect of CTRP1 on M1/M2 macrophages ratio following MI by immunofluorescence staining with M1 markers (F4/80 and NOS2) and M2 markers (F4/80 and CD206). As shown in **Figures 6E–G**, knocking down CTRP1 decreased the ratio of M1 macrophages to M2 macrophages following MI, while supplementing CTRP1 increased the ratio of M1 macrophages to M2 macrophages following MI. These results indicate that CTRP1 promotes macrophages M1 activation.

### Macrophage Depletion Reduces the Effect of CTRP1 *In Vivo*

**Figure 7:**
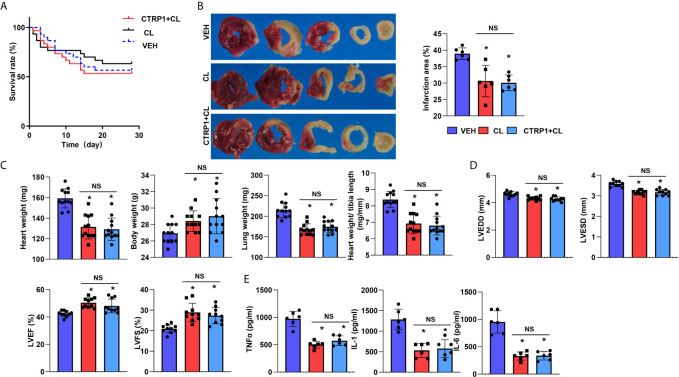
Macrophage depletion reduces the effect of CTRP1 *in vivo.* **(A)** The survival rate of mice receiving clodronate liposome (CL) injection and the human recombinant CTRP1 protein at 28 days post MI (n=30 per group). **(B)** TTC staining and quantification of the infarct area (n=6). **(C)** Heart, body, and lung weights of mice at 28 days post MI (n=12). **(D)** Echocardiography of mice at 28 days post MI (n=10). **(E)** Inflammatory cytokine levels in heart tissue post MI (n=6). *P<0.05 *vs.* the VEH group. NS, not significant.

Based on the data above, we suspected that CTRP1 exerts its effects on cardiac remodelling post MI *via* macrophages. Thus, a macrophage clearance method utilizing CLs was used *in vivo*. As shown in **Figure 7A**, the death rates in the three groups were not different. However, mice in the CL group showed a reduced infarct size compared to those in the VEH group (**Figure 7B**). Furthermore, body weights were enhanced while heart and lung weights were reduced in mice injected with CL compared with mice of the VEH group (**Figure 7C**). Cardiac dysfunction was improved by CL treatment, and inflammatory cytokine levels in heart tissue were also decreased in the CL group post MI compared with those in the VEH group (**Figures 7D, E**). However, for mice receiving both the recombinant human CTRP1 protein and CL injection, their infarct size, heart weight, lung weight, cardiac function and inflammatory cytokine levels in heart tissues did not differ from those in the CL group, and the CL group exhibited significant improvements compared with the VEH group (**Figures 7B–E**). These results suggest that CTRP1 functions *via* macrophages during the remodelling process post MI.

### CTRP1 Increases the Interaction Between Adiponectin Receptor 1 and TLR4

**Figure 8:**
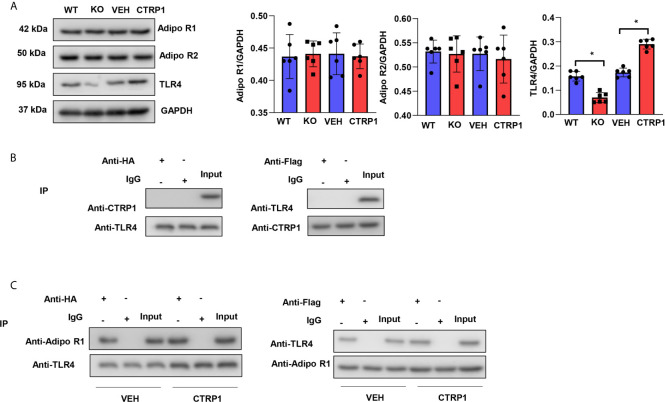
CTRP1 increases the interaction between adiponectin receptor 1 and TLR4. **(A)** The expression levels of Adipo R1, Adipo R2 and TLR4 in bone marrow-derived macrophages isolated from CTRP1 -/- mice as well as WT mice treated with CTRP1 *in vitro*. **(B)** Bone marrow-derived macrophages were cotransfected with psicoR-HA-TLR4 and psicoRFlag-CTRP1, and a coimmunoprecipitation experiment was performed. **(C)** Bone marrow-derived macrophages were cotransfected with psicoR-HA-TLR4 and psicoRFlag-Adipo R1, and a coimmunoprecipitation experiment was performed. *P<0.05. All *in vitro* experiments were repeated 3 times.

To assess how CTRP1 affects macrophages, the reported CTRP1 receptor adiponectins R1 and R2 were evaluated in isolated primary bone marrow-derived macrophages. As shown in **Figure 8A**, in both macrophages isolated from CTRP1-KO mice and WT mice treated with CTRP1 *in vitro*, the protein levels of neither Adipo R1 nor Adipo R2 were changed. We then assessed the level of TLR4, a typical PRR that accounts for the activation of macrophages during MI. As shown in **Figure 8A**, the protein level of TLR4 was reduced in macrophages isolated from CTRP1-KO mice but increased in macrophages treated with CTRP1. To investigate whether CTRP1 binds to TLR4 on the macrophage membrane, Co-IP experiments were performed and revealed that CTRP1 and TLR4 did not directly interact (**Figure 8B**); however, AdipoR1 could bind to TLR4 on macrophages, and CTRP1 enhanced the interaction between Adipo R1 and TLR4 (**Figure 8C**).

### TLR4 Knockout Counteracts the Effects of CTRP1 *In Vivo*

**Figure 9:**
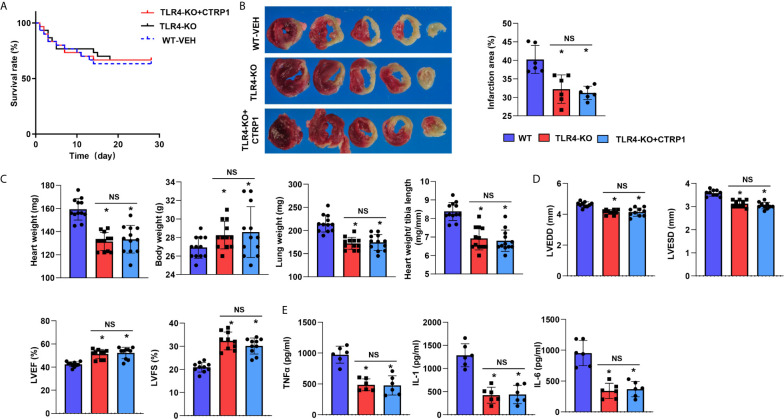
TLR4 knockout counteracts the effects of CTRP1 *in vivo.* **(A)** The survival rate of TLR4 knockout mice receiving the human recombinant CTRP1 protein at 28 days post MI (n=30 per group). **(B)** TTC staining and quantification of the infarct area (n=6). **(C)** Heart, body, and lung weights of mice at 28 days post MI (n=12). **(D)** Echocardiography of mice at 28 days post MI (n=10). **(E)** Inflammatory cytokine levels in heart tissue post MI (n=6). *P<0.05 *vs.* the WT-VEH group. NS, not significant.

TLR4-KO mice were subjected to MI and recombinant human CTRP1 protein injection, and the survival rates did not differ among the three groups (**Figure 9A**). Compared with WT control group mice, mice in the TLR4-KO group had a smaller infarct size (**Figure 9B**), higher body weight, lower heart, and lung weights (**Figure 9C**), improved cardiac function (**Figure 9D**) and reduced inflammatory cytokine levels (**Figure 9E**). TLR4-KO mice receiving the human CTRP1 protein injection also showed substantial improvements in regards to infarct size, body weight, heart weight, lung weight, cardiac function and inflammatory cytokine release compared with those in the W-VEH group (**Figures 9B–E**). These results emphasizes the role of TLR4 in the remodelling process post MI and also suggest that the role of CTRP1 was mainly rely on TLR4, as CTRP1 not exert any deteriorating effect in TLR4-KO mice.

## Discussion

In this study, we found the following: 1) CTRP1 was upregulated in both mouse hearts and heart macrophages after MI. 2) CTRP1 negatively affected the cardiac remodelling process post MI, and CTRP1 deficiency improved cardiac dysfunction, inflammation, and OS post MI. 3) CTRP1 did not affect cardiomyocytes under hypoxic conditions but rather affected the functionality of macrophages, which was consistent with the inflammation and OS in cardiomyocytes. 4) CTRP1 enhanced the interaction of TLR4 and Adipo R1 on the macrophage membrane. The clearance of macrophages or knockout of TLR4 in mice ameliorated the effects of CTRP1 post MI. Thus, all our data suggest that targeting CTRP1 is a promising therapeutic strategy for cardiac remodelling post MI.

Recently, Axel Muendlein reported that CTRP1 expression was increased in patients with stable coronary artery disease (CAD). After 8 year follow-up, they found that increased CTRP1 expression was associated with adverse cardiovascular events, including cardiac death and myocardial infarction (20). Linhui She also reported that CTRP1 expression was sharply increased in patients with CAD and positively associated with pro-inflammatory cytokines such as TNF-α and IL-6 (11). In our study, CTRP1 levels were elevated in mouse plasma and heart tissue but not adipose tissue post MI. As an adiponectin, CTRP1 is highly expressed in the heart, placenta, liver, muscle, kidney, prostate, and ovary. CTRP1 was also reported to be expressed in adipose tissue and macrophages (21). In our study, the increased CTRP1 expression post MI was not derived from cardiomyocytes but rather from macrophages either from heart native macrophages or macrophages derived from blood monocytes. And this finding was further confirmed by immunohistochemical staining of CTRP1 in infarcted heart tissue, which revealed high expression mainly in inflammatory cells. As an adiponectin, CTRP1 has controversial functions. In 2015, Parisa Shabani reported that CTRP1 was closely associated with insulin resistance in patients with nonalcoholic fatty liver disease (22). However, CTRP1 was recently found to protect against diet-induced hyperglycaemia by increasing C2C12 myotube glycolysis and fatty acid oxidation (23). CTRP1 expression was also found to be increased in patients with type 2 diabetes and to be positively associated with the levels of circulating fibroblast growth factor (FGF)21 (24), a protein that increases insulin sensitivity (25). These conflicting findings also appear in cardiovascular disease. Wu L et al. recently found that CTRP1 could protect against angiotensin II and pressure overload-induced cardiac hypertrophy (26), and CTRP1 has been reported to attenuate doxorubicin (15)- and LPS-induced cardiac injury (27). These results contradict those of a study on CTRP1 in CAD and hypertension, which suggests that CTRP1 overexpression leads to a hypertensive phenotype (28). Herein, CTRP1 knockout exerted a protective effect post MI, while the human recombinant CTRP1 protein led to a deteriorating phenotype post MI. These inconsistencies may account for the effect of CTRP1 on different cell types. While we herein found no direct role of CTRP1 on cardiomyocytes under hypoxic conditions, both Jiang W and Wu L demonstrated a direct effect of CTRP1 on cardiomyocytes *via* both angiotensin II stimulation and LPS insult. These differences may also account for distinctions between the disease models. The weight loss was observed in mice post MI, which may account for the suffering heart failure.

Macrophages are a central cell type in the pathology of both acute MI and post MI in regard to heart failure. In the acute phase, macrophages from resident heart and peripheral tissues respond to the clearance of dead cardiomyocytes (29). Later, macrophage M2 activation promotes cardiac fibrosis to maintain cardiac integrity (29). Two weeks after MI, persistent activation of M1 and M2 macrophages cause sustained cardiac inflammation and fibrosis, leading to heart failure (30). In our study, increased CTRP1 expression post MI was mainly derived from macrophages, and CTRP1 knockout induced a weak macrophage response to LPS and IFN-γ. Administration of the CTRP1 protein recovered the response of CTRP1-/- macrophages to LPS and IFN-γ, and culture medium from these macrophages could also aggravate the OS response in cardiomyocytes under hypoxic conditions. Upon macrophage clearance, the negative effects of the CTRP1 protein post MI vanished and were even reversed. TLR4 is a major molecular PRR that is expressed on the surfaces of both macrophages and cardiomyocytes (31). After activation by DAMPs, the TLR4 signal is transduced to MyD88-IRAKs-TAK1/TAB, leading to activation of NF-κB (32), the subsequent transcription of genes encoding pro-inflammatory cytokines and OS. Previous studies have confirmed that effect of TLR4 on cardiovascular disease (33, 34). Patients with reduced cardiac function have elevated TLR4 expression (33). TLR4 silencing ameliorates cardiomyocytes ischemic damage (34), cardiac injury from MI and LPS (35). The effect of CTRP1 on TLR4 has not been reported. A previous study merely found that CTRP1 functions *via* the adiponectin receptor (26). In macrophages from both CTRP1-KO and CTRP1-treated mice, the expression of neither Adipo R1 nor Adipo R2 was changed. However, we found an interaction between AdipoR1 and TLR4 in macrophages, which is consistent with a previous study on human umbilical vein endothelial cells (36). CTRP1 protein expression enhanced the interaction of adipo R1 and TLR4 in macrophages and increased the M1 activation of macrophages. Knockout of TLR4 in mice reversed the negative effects of the human recombinant CTRP1 protein post MI. These data strongly suggest that CTRP1 aggravates cardiac remodelling post MI *via* TLR4 in macrophages.

Studies have reported the protective effect of Adipo R1 activation on cardiovascular disease. By activating AdipoR1, CTRP9 mediated anti-oxidant effects in cardiac hypertrophy and failure (37). Mice lacking Adipo R1 revealed mitochondrial dysfunction in diabetic complications (38). However, study also reported that pro-inflammation response of Adipo R1 activation. Adipo R1 overexpression enhance inflammatory bowel disease in macrophage (39). In our study, we found that CTRP1 enhanced the interaction of adipo R1 and TLR4 in macrophages which promote M1 activation but not M2 activation indicating a pro-inflammation effect of CTRP1.

It is worth noting that CTRP1 may acts synergistically with other damaged or necrotic tissue and dsDNA to activate TLR4. Since as a pathogen associated molecule patterns, TLR4 can be activated by many endogenous dangerous molecules. But the detrimental function of elevated CTRP1 was confirmed in our study as it mainly targets on TLR4. The other issue is that the cardiac remodelling model we used in this study was induced by MI without reperfusion. In clinical, most acute MI patients may treat with percutaneous coronary intervention, which has a process of reperfusion. Thus, further study with an ischemia reperfusion injury model is needed to confirm the pathogenic role of CTRP1 in cardiac injury and remodelling. Besides, given the broad range of tissues in which CTRP1 is expressed and its apparently diverse roles, the development of inhibitory antibodies as well as more selective inhibitors are needed to promote clinical transformation.

In summary, our study provides a promising molecular target for the prevention and treatment of cardiac modelling post MI. Further studies aimed at determining how CTRP1 affects the interaction of adipo R1 and TLR4 in macrophages during cardiac remodelling post MI may provide valuable insight into new strategies for preventing adverse events in patients with CAD.

## Data Availability Statement

The raw data supporting the conclusions of this article will be made available by the authors, without undue reservation.

## Ethics Statement

The animal study was reviewed and approved by The Affiliated Huaian No.1 People’s Hospital of Nanjing Medical University.

## Author Contributions

YG and X-WZ contributed to the conception and design of the experiments. YG, XH, P-BG, and SW carried out the experiments. P-BG and YC analysed the experimental results and revised the manuscript. X-WZ and YG wrote and revised the manuscript. All authors contributed to the article and approved the submitted version.

## Funding

This study was supported by grants from the research fund of the Technology Development Project of Nanjing Medical University (grant no. NMUB2018148), the research start-up fund project of the Affiliated Huaian No.1 People’s Hospital of Nanjing Medical University (grant no. YGRS202006), and the Natural Science Research Program of Huai’an (HAB202025).

## Conflict of Interest

The authors declare that the research was conducted in the absence of any commercial or financial relationships that could be construed as a potential conflict of interest.
